# Taka Diastase

**Published:** 1895-10

**Authors:** Ferdinand Lascar

**Affiliations:** Pathologist to the Demilt Dispensary, etc.


### Taka Diastase.

BY FERDINAND LASCAR, PH. GR.,
Pathologist to the Demilt Dispensary, etc.

In the human system a continued waste takes place which it is necessary to provide for, and to this end man partakes of food which must contain the elements for this purpose. To bring such food products into proper form, so that they can be assimilated and taken up in the system, the digestive organs perform their functions, and these are of a mechanical and chemical order. The food needed is both animal and vegetable in nature, the latter forming by far the greater and more important part. It can truly be said that upon the proper digestion of his food, man's health, happiness, and very life depend, and progressive science has fully demonstrated the unerring truth of this. Any irregularity or fault in the process of digestion very soon becomes manifest, and dyspepsia, malnutrition, and ill health follow. As the food man partakes of is twofold, so is the process of digestion a twofold one, animal and nitrogenous foods needing an acid, while vegetable, starchy foods need an alkaline process to bring them into a soluble form ready for assimilation. The general idea about faulty digestion is that the stomach performs its duties improperly. While this, in very many instances, is undoubtedly so, the fact is, nevertheless, that in the great number of cases of impaired digestion improperly performed processes of other organs are at the bottom of the evil in failing to properly convert the starchy food partaken of.

The changing of amylaceous food into dextrose and maltose is the beginning of digestion. All will have observed that bread, crackers, or potatoes, not being sweet in themselves, very soon become so when masticated and thoroughly mixed with the saliva in the mouth, and that their taste becomes sweeter the longer this is continued. This sweet taste is due to the conversion of the hydrated starch by the action of the saliva upon it, the saliva containing an enzyme called ptyalin, which, by its presence, splits up the starch into soluble products which I will mention later on, and this splitting-up process of the starchy food even continues after it has left the stomach. Animal foods needing the acids which are found in the stomach are digested there, but acids materially interfere with the action of enzymes which cause the conversion of starch, even destroying such action altogether. For this reason it seems practically incorrect to say that the conversion of starch continues after it leaves the mouth; but nature has provided against a too soon interference of acids, because it is now well understood that acid, especially hydrochloric acid, is secreted in the stomach a considerable time after the food has arrived there, and this may be one of the reasons why the converting of starch continues after it has left the mouth.

Since medical science has thoroughly grasped the philosophy of digestion, it has been the aim, by artificial means, to supply the enzymes which digestion calls for when they do not appear to be present in a sufficient quantity or are secreted in less potent form by the digestive organs. Science has succeeded fairly well in supplying gastric and pancreatic ferments when nature lags behind; but our success has so far been only a very partial one in supplying starch-converting substances, and for this reason a new and seemingly valuable discovery in this direction at once becomes interesting.

That diastase has an identical action with ptyalin upon starch is a fact long known, and for this reason the diastase contained in malt has been employed for this purpose. Diastase is contained to a lesser or greater extent in the different extracts of malt, and in minute quantities also in fermented malt preparations. In the latter the diastatic action, however, is generally totally destroyed by the acids present. Even in the best extract of malt there is only a limited and variable amount of diastase present; and while the extract of malt will continue to play an important role as a dietetic agent, its utility as a starch-converting agent will always remain a limited one. From time to time pure diastase has been offered to the profession, but none has so far proved of a sufficient potency to recommend itself to general use. Great progress in this direction is the discovery of Mr. Takamine, a chemist of no mean ability, who acted as one of the commissioners of Japan at the Cotton Exhibition in New Orleans several years ago. At that time he showed me an extract of malt, as manufactured in Japan, very rich in diastase and nutritive properties, and which I have mentioned in a paper on the diastatic and nutritive properties of malt extracts, published in the December number, 1891, of the *Epitome of Medicine.* In that paper I warned against too great heat in the manufacture of malt extracts, as heat impairs, and is even liable to totally destroy, the diastatic action. The avoiding of all undue heat in preparing diastase may be one of the reasons why the diastase which is now manufactured by Parke, Davis & Co., under Mr. Takamine's discoveries, is so perfect in its action in converting starch into maltose and dextrose. His product is a dry powder similar in appearance to some I received from a reputable German firm years ago, but is vastly superior in potency. Since the receipt of this German preparation I have frequently had occasion to experiment with various diastases, some being named vegetable ptyalin, but in no instance have they come up to the desired standard, and failed to fill the void felt for an enzyme which will accomplish what the enzyme of saliva in a healthy individual does accomplish.

In comparing notes of experiments lately conducted with taka diastase, other available diastases, and different extracts of malt, I find that the claim of the taka diastase that it will convert a hundred times its own weight of starch into a soluble state is well authenticated, for I have succeeded in converting even fifty per cent more of starch than is claimed for it. Another point in favor of taka diastase above other similar products is the quickness of its action upon starch, for the action is almost instantaneous. To convert one hundred parts of starch into a soluble state by the action of one part of taka diastase, under proper conditions, it takes only four minutes until neither iodine test nor the microscope can detect unconverted starch. The product of converted starch with Mr. Takamine's taka diastase is to a great extent maltose. Compared with the time required by the best extract of malt to convert starch, this is certainly an excellent showing, for it took the best malt extract between seven and eight minutes to convert its own weight of starch into a soluble state, while with some other extracts of malt it took fifteen, twenty and thirty minutes to partially accomplish this end. Tests with Fehling's solution to ascertain in the converted starch products the amount of contained sugar therein were equally favorable to taka diastase.

In converting starch into a soluble state by the action of diastase, the rearranging of the molecules of starch is understood to be as follows:

Starch $(C_{12}H_{20}O_{10})$ 10 plus water, $H_2O$, are first formed into erythro dextrose and maltose.

$$(C_{12}H_{20}O_{10})_9 \text{ and } C_{12}H_{22}O_{11}$$

By the continued action of disatase further hydration of the erythro-dextrose takes place.

The erythro-dextrose further splits up into erythro-dextrose and maltose, the ultimate result being a small amount of dextrin (anchro-dextrose) and eight or nine equivalents of maltose. Since Leuch's discovery of the specific starch-converting property of saliva and its ptyaline, we have lacked an agent of sufficient potency to accomplish what good healthy saliva does, and, for the first time, we find in taka diastase a substitute of undoubted worth, which, even in the presence of a minute quantity of acid, does not cease to be potent. The ptyaline in saliva is present there in a neutral or weak alkaline state, and for this reason it suggests itself that diastase, being an analogue with the former, acts also at its best in such a state, and is incompatible with acids. I employed in the greater number of my experiments with diastase carefully washed arrow-root,—-a perfectly bland and neutral starch; but I found that starches giving a slight acid reaction on blue litmus were equally well converted by taka diastase. In testing diastase as to its potency, I would recommend that the iodine as well as the copper tests be employed, and that undue employment of heat under all circumstances should be guarded against, as heat, as already mentioned, destroys the action of diastase.

Taka diastase being a dry powder, tasteless and of no perceptible odor, can be given in very small bulk, and for this reason I think it will prove itself of value in infant feeding, where it is desirable to give starch-containing foods, provided such food would easily dissolve and the infant's saliva could be relied upon to perform that function. That the new diastase is destined to become a favorite with the profession I have no doubt, having acquainted myself with its potency in converting starch in a minimum of time into a form ready for absorption by the system, and I think it will be found the very remedy for which we have waited so long.—*Therapeutic Gazette*, July 15, 1895.

